# Targeting the Aryl Hydrocarbon Receptor: The Potential of Indole Compounds in the Treatment of Cystic Fibrosis

**DOI:** 10.3390/ijms26209876

**Published:** 2025-10-10

**Authors:** Sen Hou, Qingkun Yue, Xia Hou, Qingtian Wu

**Affiliations:** School of Basic Medicine, Jiamusi University, Jiamusi 154007, China; 18846267206@163.com (S.H.); 19545397763@163.com (Q.Y.)

**Keywords:** indole, aryl hydrocarbon receptor, cystic fibrosis, pharmacological action, drug development

## Abstract

The aryl hydrocarbon receptor (AHR), a ligand-activated transcription factor, plays a crucial role in regulating immune homeostasis, inflammatory responses, and intestinal barrier function. Indole compounds and their derivatives are ligands of AHR, which can activate the AHR signal transduction pathway and show significant regulatory potential in various inflammatory and immune diseases. Cystic fibrosis (CF) is a life-threatening autosomal recessive genetic disorder. Cystic fibrosis transmembrane conductance regulator (CFTR) dysfunction affects multiple systems throughout the body. The core of its pathological process is chronic infection, abnormal inflammation, and tissue damage caused by mucus accumulation. Exploring alternative or adjunctive therapeutic strategies targeting pathological pathways downstream of CFTR is of significant importance. The aim of the present study is to explore the multiple beneficial effects that indole compounds may exert in regulating pulmonary infection and inflammation, repairing intestinal barrier function, and regulating immune homeostasis in CF patients by activating the AHR signaling pathway. Additionally, this study discusses the risks and challenges associated with developing indole compounds as CF drugs, offering a novel research approach distinct from traditional CFTR modulators for creating new CF therapeutics.

## 1. Introduction

Cystic fibrosis (CF) is an autosomal recessive genetic disorder caused by mutations in the cystic fibrosis transmembrane conductance regulator (CFTR) gene. These mutations lead to functional defects in the CFTR chloride channel, which in turn affect the respiratory, digestive, and immune systems of patients. CF-targeted drugs such as CFTR modulators are costly and not applicable to all CF mutation types, leaving a significant number of patients unable to benefit from them. Therefore, the development of new CF drugs still requires continuous exploration.

Recent studies have demonstrated that the aryl hydrocarbon receptor (AHR), as a key receptor for sensing environmental, dietary, and microbial signals, plays a central role in maintaining immune homeostasis, alleviating inflammation, enhancing epithelial barrier function, and regulating microbiota-host interactions. AHR can be activated by indole compounds. The indole skeleton is considered an ideal structure for targeting AHR. Through further chemical optimization of this skeleton, it is possible to achieve highly efficient, potent, and selective targeting of AHR, thereby enabling its use in treating related diseases. Of particular note is that AHR activation effectively modulates inflammatory responses and enhances epithelial barrier function, precisely targeting the core pathological changes affecting multiple systems in CF patients. Indole compounds can be produced through the metabolism of dietary tryptophan (Trp) by gut microbiota. Multiple bacterial strains isolated from the skin of healthy individuals can metabolize Trp to generate indole compounds. Certain strains, such as *Micrococcus luteus*, even possess the ability to synthesize tryptophan de novo [1]. Beyond natural metabolites, indole derivatives designed and synthesized through medicinal chemistry approaches may also serve as effective AHR modulators, offering novel pathways for drug discovery. For example, novel heterocyclic compounds synthesized based on the indigo carmine skeleton demonstrated significant anticancer activity in vitro, suggesting their structural potential [2]. Therefore, the systematic exploration and design of indole compounds and their derivatives targeting the AHR pathway have opened up a novel direction for developing highly effective and low-toxicity CF therapeutic agents.

This review aims to thoroughly explore the molecular pathophysiology and current clinical treatment landscape of CF, with a particular focus on research advances regarding indole compounds and the AHR pathway in regulating immune-inflammatory responses, defending against pathogen infections, and maintaining the integrity of intestinal and pulmonary barriers. It systematically evaluates the development prospects, risks, and challenges associated with indole compounds as novel therapeutic strategies or adjunctive medications for CF, providing a novel theoretical foundation and drug development direction to overcome current limitations in CF treatment.

## 2. CFTR

### 2.1. CFTR Function and Dysfunction

The CFTR protein is a member of the ATP-binding cassette (ABC) transporter superfamily. Its structure includes two membrane-spanning domains (MSD1 and MSD2), two nucleotide-binding domains (NBD1 and NBD2), and a regulatory domain (R domain) [3]. CFTR is expressed on the epithelial cell membranes of the gastrointestinal tract, sweat glands, exocrine pancreas, and various epithelial and non-epithelial tissues [4]. Its main function is to mediate the transmembrane transport of the Cl^−^ [5], and it also participates in the transcellular transport of the HCO_3_^−^ [6]. A key role of CFTR is in regulating the volume, salt content, and pH of airway surface liquid (ASL) and other epithelial secretions [7,8]. Furthermore, CFTR regulates other channels like the epithelial sodium channel (ENaC) and is involved in maintaining epithelial tight junctions (TJs), controlling secretion acidity, sphingosine-1-phosphate transport, and inflammatory signaling [9,10].

Loss of CFTR function disrupts epithelial ion transport, initiating a cascade of events: Cl^−^ secretion disorder reduces the infiltration of water into the lumen. Enhanced ENaC activity leads to excessive absorption of Na^+^, further exacerbating mucus dehydration [11]. Consequently, secretions like respiratory mucus, pancreatic juice, and intestinal fluid become abnormally thick and obstructive due to water deficiency [12]. The respiratory system is the one most affected by CF and is also the main cause of death for patients [13,14]. Thick airway mucus obstructs breathing, impairing gas exchange and causing cough and dyspnea [15]. It also promotes the colonization of bacteria such as *Pseudomonas aeruginosa*, leading to chronic infection and inflammation. This creates a vicious cycle of airway obstruction, chronic infection, and inflammation, resulting in progressive lung damage [16]. CF also impacts the digestive system. Impaired CFTR function leads to insufficient bicarbonate secretion, failing to neutralize gastric acid and creating an unsuitable pH for digestive enzymes [17]. At the same time, bicarbonate deficiency causes intestinal mucus obstruction and meconium ileus [18]. Furthermore, approximately 85% of CF patients have severely damaged extracellular secretory glands of the pancreas at birth [18]. The reproductive system is affected as well. CFTR is expressed in both male and female reproductive organs. Male patients with CF are often accompanied by congenital bilateral absence of the vas deferens (CBAVD), congenital unilateral absence of the vas deferens (CUAVD), or infertility due to azoospermia [19]. Female patients experience a significant decline in fertility due to CFTR mutations in the reproductive tract epithelial cells [20].

### 2.2. CFTR Mutation Classes

Following synthesis on the ribosome, the nascent CFTR polypeptide chain enters the endoplasmic reticulum (ER), where it undergoes core glycosylation, forming the B Band [21]. Properly folded protein is transported to the Golgi for complex glycosylation, yielding the mature, functional C Band form of the protein, which acts as an ion channel [22]. Misfolded mutant proteins are recognized by the endoplasmic reticulum quality control (ERQC) system and degraded via the ubiquitin-proteasome pathway [23].

**Figure 1:**
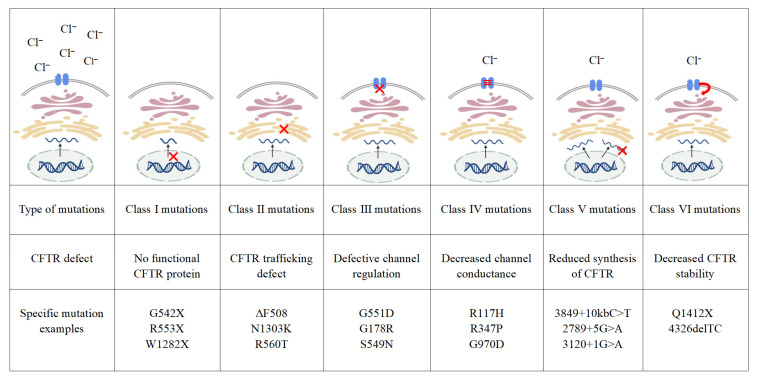
Classes of CFTR mutations [24]. Class I mutations result in no protein production, red symbol in class I mutations indicate truncated, nonfunctional mRNA produced in the nucleus. Class II mutations result in misfolded proteins being degraded via the proteasome pathway, red symbol in class II mutations indicate misfolded proteins failing to pass through the endoplasmic reticulum quality control system. Class III mutations impair channel opening, red symbol in class III mutations indicate the CFTR ion channel on the cell membrane failing to open. Class IV mutations show reduced conduction, wave lines in class IV mutations indicate reduced chloride ion transport efficiency. Class V mutations cause a significant reduction in mRNA or protein levels, or both, red symbol in class V mutations indicate unstable or nonfunctional mRNA produced in the cell nucleus. Class VI mutations reduce the stability of ion channels on the cytoplasmic membrane, red arrow in class VI mutations indicate that CFTR proteins reaching the cell membrane are prone to degradation; created in BioGDP. CFTR: cystic fibrosis transmembrane conductance regulator.

CFTR mutations are categorized into six classes based on their functional consequences (Figure 1) [24]. CF was historically considered predominant in Caucasians, with F508del-CFTR representing the most prevalent mutation [25]. Research on CFTR in China has only gained momentum in recent years. Following CF’s inclusion in China’s first list of rare diseases (2018), studies revealed significant differences in CFTR mutation spectra between Chinese and Western populations. Common mutations among Chinese CF patients include G970D (Class IV mutation causing reduced functional protein expression) and I1023R (which may impair protein processing/trafficking and reduce glycosylation without affecting channel open probability) [26]. The common mutant type in Western populations is F508del-CFTR (Class II mutation, where misfolded proteins are degraded by the ubiquitin-proteasome system and cannot reach the cell membrane to perform their function).

### 2.3. Therapeutic Advances in CF

Although CF cannot be cured, comprehensive treatment such as nutritional support, antibiotics, CFTR modulators, and stem cell therapy can prolong the life of patients [27,28]. Internationally, various small-molecule modulators have been developed for different mutation classes. The CFTR potentiator Ivacaftor as monotherapy targets class III gating mutations (e.g., G551D) but applies to about 5% of CF patients [29]. Dual therapy Lumacaftor/Ivacaftor is being superseded by triple regimens due to limited efficacy and hepatotoxicity concerns [30]. The triple-combination Elexacaftor/Tezacaftor/Ivacaftor, approved by the US FDA and other regulators, treats patients with at least one F508del mutation [31]. However, its efficacy is limited for some rare mutations and digestive manifestations. When modulators are insufficient, supportive care for affected organs is essential. In addition to modulators, gene therapy has also entered the clinical trial stage. The emergence of CFTR modulators and gene therapy has revolutionized the treatment model for CF. However, it is difficult to obtain clinical trial data for rare mutations, which cannot cover all CF patients. Developing new CF drugs is therefore still necessary.

## 3. AHR

### 3.1. AHR Signaling Pathway

AHR is a ligand-activated transcription factor containing a basic helix-loop-helix (bHLH) domain and a Per-ARNT-Sim (PAS) domain [32]. It is widely expressed in human and animal tissues and is structurally conserved across species. In the non-activated state, AHR resides in the cytoplasm as part of a complex with chaperone proteins, including heat shock protein (HSP) 90, X-associated protein 2 (XAP2), and the co-chaperone p23 [33].

**Figure 2:**
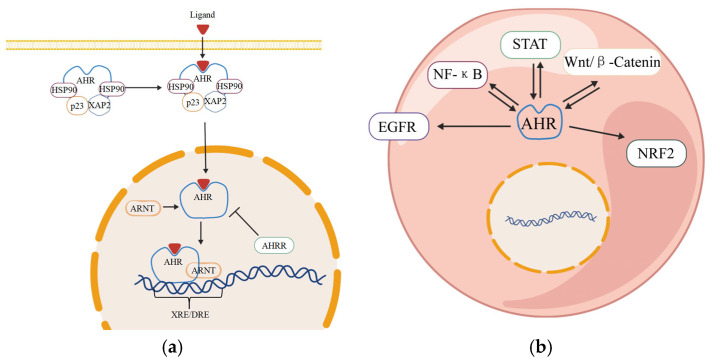
AHR signaling pathway; created in BioGDP. (**a**) AHR classic signaling pathway [34]; (**b**) AHR non-classical signaling pathway [35]. AHR: aryl hydrocarbon receptor. HSP90: heat shock protein 90. XAP2: X-associated protein 2. ARNT: aryl hydrocarbon receptor nuclear translocator. AHRR: aryl hydrocarbon receptor repressor. EGFR: epidermal growth factor receptor. NF-κB: nuclear factor kappa-B. STAT: signal transducer and activator of transcription. NRF2: nuclear factor erythroid 2-related factor 2.

During the classical AHR signaling pathway, the cytoplasmic AHR complex protein undergoes conformational changes after binding with ligands, separates from the chaperone proteins, and exposes the N-terminal nuclear localization signal to enter the nucleus. Inside the nucleus, AHR dimerizes with the aryl hydrocarbon receptor nuclear translocator (ARNT). The AHR-ARNT complex binds to DNA sequences containing the aryl hydrocarbon receptor elements to regulate the expression of downstream target genes [34] (Figure 2a).

AHR also has non-classical signaling pathways that function by cross-referencing with other signaling pathways, such as nuclear factor kappa-B (NF-κB) and signal transducer and activator of transcription (STAT) [35] (Figure 2b). AHR and NF-κB regulate common target genes, including certain cytokines. For instance, 2,3,7,8-tetrachlorodibenzo-p-dioxin (TCDD) upregulates interleukin (IL)-1β, IL-6, and IL-8 expression via AHR, and this effect is mediated through NF-κB and extracellular regulated protein kinase (ERK) signal cascade [36]. AHR binds to RELA or RELB within the NF-κB family to regulate the transcriptional activity of downstream target genes [37], thereby inhibiting IL-6 and negatively regulating the inflammatory response [38]. Under the stimulation of TCDD and lipopolysaccharide (LPS), AHR and RELB recruitment to the IL-22 promoter region is enhanced, promoting IL-22 expression and production [39]. AHR can also engage in cross-talk with NF-κB through other signaling nodes. For instance, acteoside modulates NF-κB RELA and nuclear factor erythroid 2-related factor 2 (NRF2) protein expression by inhibiting the AHR signaling pathway and its downstream gene expression, thereby forming an AHR-NRF2-NF-κB signaling axis. This axis jointly coordinates oxidative and inflammatory responses, improving renal fibrosis [40]. Interactions between AHR and STAT pathways are also documented. Activated AHR can induce phosphorylation of Janus kinase-2 (JAK2) and its downstream effector STAT3 [41]. Indole derivatives activate AHR to promote IL-22 secretion, which in turn stimulates STAT3 phosphorylation, enhancing epithelial cell proliferation and restoring intestinal barrier integrity [42]. Additionally, AHR influences STAT protein activity and stability by regulating the expression of suppressor of cytokine signaling (SOCS) family proteins, such as SOCS2 [43].

Prolonged AHR activation can be harmful. The feedback regulation and termination mechanism of its signals enable it to achieve rapid metabolism. Upon completing its transcriptional activation function, AHR undergoes rapid degradation through the ubiquitin-proteasome pathway to regulate its cytoplasmic levels [44]. This ligand-dependent rapid degradation represents a key mechanism for desensitizing AHR signaling, thereby limiting the duration and intensity of the signal. Activated AHR induces the expression of target genes within the cytochrome P450 family 1 subfamily A member 1 (CYP1A1) and cytochrome P450 family 1 subfamily B member 1 (CYP1B1). These enzymes also metabolize excessive AHR ligands and regulate their own signal activity through feedback, thereby shortening the duration of AHR signal transduction [45]. The aryl hydrocarbon receptor repressor (AHRR) is one of the target genes of AHR. It competitively binds to ARNT, forming the AHRR-ARNT dimer. This dimer binds to the XRE region of the AHR target gene but does not activate transcription, thereby effectively inhibiting AHR-mediated transcriptional activation [46].

### 3.2. Activation of AHR by Indole Compounds

Dietary, microbial, and metabolic signals modulate AHR signaling, thereby regulating diverse biological processes [47,48]. Currently known indole ligands primarily fall into four major categories: Endogenous Trp metabolites produced within the human body; indoles generated by the human microbiome, which occupy an intermediate position between endogenous and exogenous sources; exogenous indoles derived from dietary or pharmaceutical sources; and rationally designed novel indole AHR ligands [49]. Trp is an essential amino acid obtained through dietary intake, and it regulates the balance between anti-inflammatory and pro-inflammatory cytokines [50]. In the body, Trp is metabolized through three primary pathways: kynurenine (Kyn), serotonin, and the microbial indole pathway [51]. The gut microbiota directly metabolize Trp into indole and its various derivatives. Structurally, indole is an aryl heterocyclic compound with a benzene ring and a nitrogen-containing pyrrole ring. The hydrogen atoms in the pyrrole ring can shift positions to form three isomers. This structural characteristic endows indole compounds and their derivatives with rich chemical activity and the ability to interact with other compounds [52]. Acting as important signaling molecules, indole derivatives are involved in the development and progression of diseases in the digestive, nervous, and urinary systems [53], and play a key role in maintaining gut microbiota balance and host health [54].

As endogenous AHR ligands, indole compounds derived from Trp metabolism help regulate intestinal mucosal immunity, barrier function, and homeostasis. Research has found that indole compounds produced by microbial decomposition of Trp activate the AHR signaling pathway, upregulate the expression of IL-22 in local intestinal tissues, and participate in the regulation of intestinal inflammatory processes [55,56]. For instance, 6-formylindolo [3,2-b]carbazole (FICZ) activates AHR to inhibit DSS-induced intestinal damage and inflammatory cell infiltration, thereby alleviating colitis in mice [57]. AHR activation participates in downstream homeostatic mechanisms, promotes intestinal epithelial renewal, protects the integrity of the intestinal barrier, and regulates many cell types, including intraepithelial lymphocytes and T helper cell (Th) 17 cells [58]. Similarly, indole-3-carbinol (I3C) and its derivatives influence T cell differentiation via AHR, increasing regulatory T cell (Treg) generation while reducing Th cell numbers, which helps mitigate intestinal inflammation [59].

## 4. AHR with CF

AHR signal transduction plays a potentially important role in maintaining immune homeostasis, reducing the risk of infection, and improving lung function in CF patients.

### 4.1. Pulmonary Infection and Inflammation

Pulmonary disease is the most prominent clinical manifestation of CF and also the main cause of death for CF patients. CFTR defects cause ASL dehydration and viscous mucus retention, leading to chronic infection and inflammation [60]. *Lactobacillus* species metabolize Trp to produce indole-3-lactic acid (ILA), which stimulates type 3 innate lymphoid cells (ILC3s) to produce IL-22, which upregulates the expression of antimicrobial peptide AMP-17 and resists the colonization of pathogens such as *Candida albicans* [61]. *Pseudomonas aeruginosa* is the most common pathogen in the respiratory tract of CF patients. Infection with this bacterium exacerbates the clinical symptoms of CF patients, and the dehydrated mucus environment promotes the formation of its biofilm [62]. Its strong antibiotic resistance further complicates treatment [63]. AHR recognizes and binds phenazine virulence factors produced by *Pseudomonas aeruginosa*. In the zebrafish model, AHR activation changes with bacterial concentration, suggesting it senses population density and activates host defenses at high bacterial loads [64]. Indole-3-carbaldehyde (ICA) as an endogenous agonist of AHR, exerts dual effects of enhancing the epithelial barrier and antimicrobial activity. Based on this, researchers developed an inhalable dry powder of ICA, which demonstrated excellent antibacterial activity against pathogens such as *Pseudomonas aeruginosa*. Animal experiments confirmed that ICA, as an anti-inflammatory agent, has dual therapeutic advantages of preventing pulmonary inflammation and alleviating pulmonary infection [65].

CF patients’ lungs are also more susceptible to viral infections due to mucus accumulation, impaired ciliary function, and chronic inflammation [66]. Viral infection significantly inhibits the AHR signaling pathway, while consuming foods rich in indole compounds, such as cruciferous vegetables, can activate the AHR-Apelin signaling pathway in lung endothelium, maintain lung barrier function, and alleviate lung injury [67]. This implies that dietary indoles may modulate AHR activity to improve inflammatory diseases in multiple organs. Patients with CF have chronic inflammation in the lungs, and functional AHR is expressed in alveolar epithelial cells (AECs). Activation of the AHR signaling pathway by FICZ significantly inhibits the expression of inflammatory cytokines in LPS-induced mouse lung tissue and bronchoalveolar lavage (BAL), and enhances the barrier function of primary mouse AECs, as evidenced by increased transepithelial electrical resistance (TEER). Following LPS-induced inflammatory injury, AHR activation also inhibits the decrease in TEER [68].

### 4.2. Intestinal Barrier and Microbiota

CFTR mutations impair chloride transport and affect intestinal TJs expression and function, compromising barrier integrity [69]. In the intestinal tract, *Lactobacillus* metabolizes Trp to produce ILA, which enhances Trp metabolism and increases indole-3-propionic acid (IPA) and indole-3-acetic acid (IAA) levels [70]. In both DSS-induced colitis mouse models and IL-10^−/−^ spontaneous colitis mouse models, IPA enhances the integrity of the epithelial barrier and alleviates colitis by activating the AHR signaling pathway [71]. After DSS-induced colitis in mice, the expression levels of ZO-1, claudin-1, and occludin in the mouse intestine were significantly decreased, while FICZ treatment restored their expression levels [72]. *Parabacteroides distasonis* enhances Trp metabolism and increases indole-3-acrylic acid (IA), activating AHR and upregulating IL-22 to promote barrier repair in diabetic rats. In Caco-2 cells, IA supplementation strengthened barrier-related protein expression through AHR activation [73]. The changes in the intestinal microecology of CF patients lead to their inflammatory environment, which is transmitted to other parts through mucosal immunity, especially to the lungs via the “lung-gut” axis [74]. This suggests that modulating intestinal homeostasis may alleviate CF symptoms. Dietary nutrients metabolized by gut microbes help maintain intestinal permeability and homeostasis via AHR activation [75]. The activation of the Trp-AHR pathway maintains the intestinal mucosal barrier function to a certain extent. The above evidence indicates that indole compounds produced by Trp metabolism improve the damaged intestinal barrier by activating AHR.

CF patients show significantly altered diversity and composition of gastrointestinal microbiota compared to healthy individuals [76,77]. This dysbiosis of the microbiota further affects the host’s immunity and metabolism and is an important driving factor for intestinal immune damage [78]. *Lactiplantibacillus plantarum* CGMCC9513 restores the imbalance of the intestinal microbiota in mice with ulcerative colitis by activating AHR and upregulating the levels of beneficial bacteria such as *Lactobacillus* [79]. Analysis of the gut microbiota in patients with CF revealed significant differences in the composition of *Lactobacillus* compared to healthy controls, suggesting that supplementing indole compounds or probiotics may help restore gut microbiota homeostasis in CF patients.

### 4.3. Immune Homeostasis

Studies in CF pig models reveal altered lung immune cell composition at birth, including increased monocytes and immature neutrophils, consistent with reduced CD16 expression in myeloid cells of children with CF [80]. Immature immune cells may exacerbate the vicious infection-inflammation cycle in CF patients, and the existing CFTR modulators are unable to fully correct the impact of mutations on the immune system [81]. Dendritic cells (DCs) are the key bridge connecting innate immunity and adaptive immunity. AHR regulates the differentiation and function of DCs. The AHR antagonist Stemregenin 1 (SR1) inhibits AHR activity, promoting the differentiation of CD34^+^ cells into myeloid and plasma cell-like DCs and increasing the secretion of interferon-α (IFN-α), IL-12, and tumor necrosis factor (TNF). Conversely, the activation of AHR by FICZ will promote the differentiation of monocytes into DCs and macrophages [82]. Persistent toll-like receptor (TLR) 4 activation in lung DCs of CF patients increases secretion of IL-6 and TNF-α, exacerbating Th17 responses. The AHR agonist 2-(1′-H-indole-3′-carbonyl)-thiazole-4-carboxylic acid methyl ester (ITE) modulates production of Th1-associated IL-12 and Th17-associated IL-6, IL-22, and transforming growth factor-β (TGF-β) [34], indicating that AHR activation may help normalize CF-related immune dysregulation.

## 5. Indole Compounds and Derivatives with CF

### 5.1. Anti-Inflammatory and Antibacterial Mechanisms of Indole Compounds

Many indole compounds act as AHR ligands to regulate inflammatory factors, modulate gut microbiota, and enhance barrier function (Table 1).

ICA reduces the release of proinflammatory cytokines such as IL-1β, IL-6, and TNF-α by inhibiting the TLR 7/NF-κB pathway. In the mouse model infected with respiratory syncytial virus (RSV), ICA also downregulates IFN-α secretion, balancing antiviral and proinflammatory cytokine effects to maintain viral clearance capacity while avoiding immune damage [83]. This anti-inflammatory mechanism suggests it may exert a regulatory effect on pulmonary inflammation in CF patients. Abnormal changes in the gut microbiota of CF patients lead to elevated inflammation levels. In vitro experiments demonstrate that indole induces intestinal epithelial cells to increase the expression of TJs and the anti-inflammatory cytokine IL-10, while reducing levels of the pro-inflammatory cytokine IL-8. Animal studies reveal that indole reduces *Escherichia coli* adhesion capacity, enhancing host resistance to pathogenic microorganisms while simultaneously suppressing inflammatory responses [84]. The traditional Chinese medicine Wuji Pill can regulate the gut microbiota, increase the abundance of *Lactobacillus*, promote the production of IAA and IA, thereby reducing colonic inflammation and restoring the expression of intestinal barrier proteins [85]. CF patients exhibit symptoms of gut microbiota dysbiosis and impaired intestinal barrier function. The reduction of IAA may aggravate intestinal inflammation and barrier dysfunction. IPA enhances the intestinal barrier by increasing mucins (MUC2 and MUC4) and goblet cell secretory products trefoil factor 3 (TFF3) and resistin-like molecule β (RELMβ). Additionally, IPA can reduce the inflammatory factors induced by LPS [86]. Patients with inflammatory bowel disease (IBD) exhibit significantly reduced levels of IPA in their stool. Researchers have found that IPA promotes the apoptosis of Th1/Th17 cells in the intestinal mucosa and enhances the intestinal mucosal barrier by binding to HSP70. The above findings indicate that IPA plays a key role in alleviating intestinal mucosal inflammation [87].

Dietary indole compounds such as I3C naturally occur in cruciferous vegetables, exhibiting antioxidant and anti-inflammatory properties, and can prevent alcohol-induced alterations in TJs. I3C polymerizes in acidic environments to form derivatives such as 3,3′-diindolylmethane (DIM), which exhibits anti-inflammatory and anti-cancer properties. In the mouse model of liver injury, DIM suppresses reactive oxygen species (ROS) by downregulating NF-κB, thereby reducing pro-inflammatory mediators [88]. The anti-inflammatory properties of I3C and DIM may modulate pulmonary inflammation in CF patients.

**Table 1:** Indole ligands for AHR. AHR: aryl hydrocarbon receptor. CF: cystic fibrosis. DIM: 3,3′-diindolylmethane. FICZ: 6-formylindole [3,2b]carbazole. IPA: indole-3-propionic acid. I3C: indole-3-carbinol. ICA: indole-3-carbaldehyde. IAA: indole-3-acetic acid. IA: indole-3-acrylic acid. DSS: dextran sulfate sodium salt. LPS: lipopolysaccharide. EC: endothelial cell. RSV: respiratory syncytial virus. HFD: high-fat diet.

Ligand	Structure	Source	Model	Dose	Effect	Stage
DIM	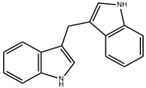	Cruciferous vegetables	Liver-damaged mice [88]	2.5–10 mg/kg (Subcutaneous injection)	Reduce inflammatory factors, anti-inflammatory effects	Preclinical trials
FICZ	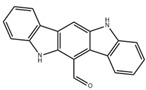	Photo-oxidation	DSS-induced colitis in mice [57]	1 μg/day (Intraperitoneal injection)	Reduce intestinal inflammation, enhance intestinal barrier	Preclinical trials
			LPS-induced acute lung injury in mice [68]	1 μg (Intranasal administration)	Reduce inflammatory factors, enhance pulmonary barrier function	
IPA	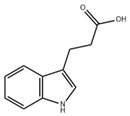	Microbiota metabolism	LPS-induced inflammation in Caco-2/HT29 co-cultured cells [86]	0.05–0.5 mM (In vitro processing)	Anti-inflammatory effect	Preclinical trials
			DSS-induced colitis in mice [89]	50 mg/kg/day (Oral gavage)	Enhance intestinal barrier	
I3C	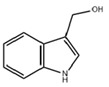	Cruciferous vegetables	Alcohol-induced alcoholic liver injury in mice [90]	40 mg/kg/day (Oral gavage)	Antioxidant, anti-inflammatory, anti-apoptotic effects on the gut-liver-adipose tissue axis	Preclinical trials
			Influenza virus infection in EC^ΔAHR^ mice [67]	1000 ppm I3C (Added to the diet)	Maintain lung barrier function, reduce lung injury	
ICA	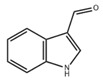	Microbiota metabolism	RSV-induced inflammation in RAW264.7 cells [83]	10–50 μM (In vitro processing)	Anti-inflammatory effects, reduce the production of pro-inflammatory cytokines	Preclinical trials
			Mice infected with *Aspergillus fumigatus spores* via intranasal instillation [91]	18 mg/kg (Intranasal delivery, oral administration) 4.5 mg/kg (Blowing into the lungs)	Possess antibacterial activity, prevent lung inflammation, alleviate lung infections	
IAA	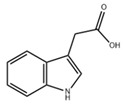	Microbiota metabolism	HFD-induced obese mice [92]	50 mg/kg/day (Oral gavage)	Reduce systemic inflammation, regulate gut microbiota composition, increase the abundance of beneficial bacteria	Preclinical trials
IA	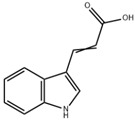	Microbiota metabolism	DSS-induced colitis mice [93]	1 × 10^9^ CFU/day (*Lactobacillus reuteri* oral gavage)	Strengthen intestinal epithelial barrier, reduce inflammation	Preclinical trials

### 5.2. Indole Compounds Related to CF

*Pseudomonas aeruginosa* usually colonizes in the lungs of CF patients. Most strains of *Pseudomonas aeruginosa* isolated from CF patients produce high levels of Kyn. Researchers have experimentally demonstrated that Kyn promotes bacterial survival and enables *Pseudomonas aeruginosa* to evade innate immune responses by scavenging reactive oxygen species generated by neutrophils [94]. Indole serves as a key volatile organic compound distinguishing CF patients from healthy individuals. CFTR dysfunction in CF patients leads to altered intestinal pH and slowed intestinal motility. Combined with long-term antibiotic therapy, this predisposes patients to gut microbiota imbalance, promoting the proliferation of indole-producing bacteria such as *Escherichia coli*. This gut dysbiosis may lead to indole and its metabolites produced in the intestine entering the bloodstream through the compromised intestinal barrier, thereby affecting the pulmonary environment via the “lung-gut axis” and being reflected in exhaled breath [95]. Inhaled indole not only serves as a marker to distinguish CF patients from healthy individuals, but within the CF patient population itself, exhaled indole levels are not constant. Changes in its concentration correlate with disease severity: higher exhaled indole levels in CF patients indicate poorer lung function. Researchers also validated through machine learning algorithms that the concentration of indole in exhaled breath serves as a reliable indicator for determining whether patients are infected with *Pseudomonas aeruginosa* [96]. The above studies suggest that indole may serve as a biomarker for monitoring disease progression and gut microbiota dysbiosis in CF patients. Elevated indole levels in the exhaled breath of CF patients do not contradict its use as a therapeutic agent. Endogenous high levels indicate an imbalance across the entire metabolic pathway, serving as both a consequence and marker of the disease state. This overall environment of imbalance is detrimental. In contrast, exogenous therapeutic supplementation involves the selective and controlled introduction of specific beneficial molecules aimed at restoring disrupted physiological equilibrium.

### 5.3. Indole Compounds and Their Derivatives as Candidate Therapeutics for CF

Evidence from preclinical to clinical studies supports the potential of indole compounds and their derivatives as CF therapeutics.

#### 5.3.1. Preclinical Experimental Evidence

ICA is a novel therapeutic agent that employs a targeted delivery system to achieve multi-mechanism treatment of CF. Rather than directly restoring CFTR function, ICA intervenes in the pathological progression of CF by modulating the core target AHR. Researchers formulated ICA into a dry powder via spray drying. Inhaled ICA dry powder alleviated lung inflammation in CF mice, with targeted lung delivery reducing systemic exposure. Pharmacokinetic data indicate undetectable AHR activation in other organs, thereby lowering the risk of systemic side effects. In CF mice with intestinal *Candida albicans* infection, intraperitoneal administration of enteric microparticle-encapsulated ICA upregulated intestinal TJs expression, enhanced barrier function, increased antimicrobial peptide levels, and modulated gut microbiota composition by promoting beneficial bacteria like *Lactobacillus*. Oral ICA also improved lung inflammation, indicating systemic regulation via the gut-lung axis and a potential to reduce underlying infection and inflammation. In vitro organ culture further confirmed that ICA activates AHR and its downstream target genes. Research indicates that targeted delivery of ICA effectively alleviates pulmonary and intestinal inflammation in CF mice, restoring immune and microbial homeostasis. This provides preclinical evidence for developing safe and effective indole compounds as CF therapeutics [91]. Indole compounds, as therapeutic postbiotics, can achieve tissue-specific delivery through drug delivery systems, offering broad prospects in the treatment of CF [97].

#### 5.3.2. Clinical Translational Basis

A randomized, double-blind, placebo-controlled crossover clinical study conducted by Rueda GH et al. provided clinical data on the action of indole compounds on the AHR pathway. This study confirmed that oral administration of L-Trp to healthy volunteers significantly elevated levels of indole metabolites in serum and urine and successfully activated the AHR signaling pathway in the small intestine [98]. This study confirmed the in vivo bioactivity of indoles as AHR agonists and assessed their clinical safety, providing a crucial reference for subsequent clinical trial design and establishing a foundation for translation.

#### 5.3.3. Indole-Based CFTR Modulators

**Figure 3:**
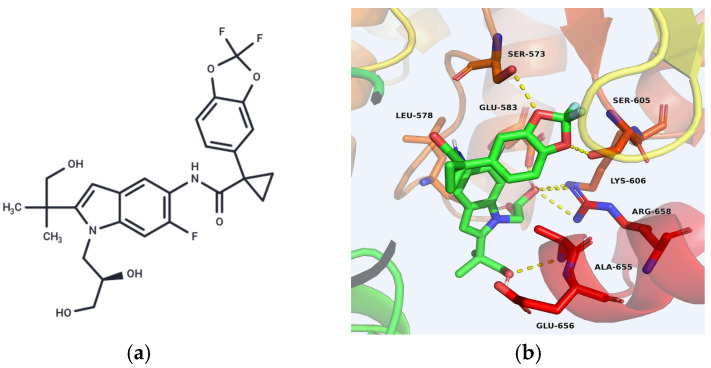
(**a**) Structure of Tezacaftor. (**b**) Key target interaction of Tezacaftor with CFTR [100].

Tezacaftor is a current CFTR modulator. As part of the triple-combination Elexacaftor/Tezacaftor/Ivacaftor, it is indicated for CF patients with at least one F508del mutation. Tezacaftor and other CFTR modulators can position the CFTR protein correctly on the cell membrane surface, regulate ion channel formation, and enhance ion channel transport function on the cell membrane, thereby repairing the protein misfolding caused by CFTR mutations [99]. The molecular structure of Tezacaftor contains an indole ring as its core (Figure 3a). Structural biology studies show that the indole moiety binds the MSD1 domain of CFTR, enhancing its stability during early biosynthesis and preventing degradation (Figure 3b). This binding stabilizes the MSD1 domain during the early stages of CFTR protein biosynthesis, preventing its degradation [100].

#### 5.3.4. Future Development Prospects

**Figure 4:**
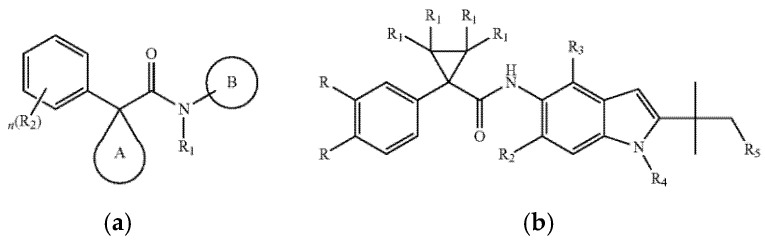
(**a**) Structure of formula I [101]. (**b**) Structure of formula II [101]. R_1_–R_5_ are different substituents.

Beyond approved drugs, innovative research continues. For instance, an invention patent by Ruah, S.S.H et al. discloses a new class of indole-based CFTR modulators. These compounds feature an indole core scaffold (Figure 4). The indole ring serves as the core skeleton, and the activity, selectivity, and pharmacokinetic properties of the compounds are regulated by introducing diverse substituents onto the indole ring. Preclinical functional studies using membrane potential measurement techniques demonstrate that these compounds effectively enhance chloride transport mediated by the F508del-CFTR mutant, confirming their potential to correct the functional defects of CFTR. The patent also outlines their suitability for various formulations and potential for use in combination therapies, highlighting significant development potential [101].

The research team led by Son JH selected spiro [piperidine-4,1-pyrido [3,4-b] indole] as the starting point for drug development. The indole moiety in this compound serves as a key component. Through conformational control and structural optimization, a novel class of CFTR modulators, termed co-enhancers, was developed. These compounds synergize with existing enhancers such as VX-770 and GLPG1837 to enhance chloride channel function in CFTR mutant variants. The specific functional groups on the indole ring and the overall three-dimensional conformation of this compound directly determine whether the molecule can effectively restore CFTR protein function. Researchers have demonstrated the efficacy of this co-enhancer against several CFTR NBD2 variants [102].

The research team led by Brindani N identified novel CFTR enhancers featuring a 2,3,4,5-tetrahydro-1H-pyridino [4,3-b] indole structure through high-throughput screening. Following systematic structure-activity relationship studies, they ultimately obtained lead compound 39, which exhibits excellent activity, exhibiting favorable in vitro drug-like properties and oral bioavailability. Cellular and animal studies confirmed its effective correction of CFTR channel dysfunction caused by F508del and G551D mutations [103].

The above findings introduce a novel chemical class to the existing category of CFTR enhancers and may expand the current therapeutic options for treating CF. It also suggests that indole can serve as a rational design template for drug development, demonstrating the advantages and promising prospects of bioactive heterocycles containing indole structures in the development of CF therapeutics.

## 6. Risks and Challenges

### 6.1. AHR Activation and Tumor Immune Escape Risk

While indole compounds show therapeutic potential in anti-inflammatory and immune tolerance via AHR activation, their long-term use requires caution due to possible side effects, including increased infection susceptibility and tumor immune escape. As noted, AHR interacts with the NF-κB and STAT signaling pathways. In many cases, AHR activation inhibits the NF-κB signaling pathway, which can alleviate inflammation. However, within the tumor microenvironment, it weakens the antitumor immune response, thereby promoting tumor immune escape. For example, IL4I1-generated Trp metabolites activate AHR and suppress adaptive immunity, correlating with poorer survival in glioblastoma patients and accelerated chronic lymphocytic leukemia (CLL) progression in mice [104]. STAT3 is a well-known oncogene whose sustained activation promotes tumor cell proliferation, survival, and invasion. In cancer-associated fibroblasts (CAFs), Kyn activates the AHR, leading to the activation of Protein Kinase B (AKT) and STAT3 signaling pathways, thereby promoting renal carcinoma progression and drug resistance [105]. In certain circumstances, AHR activation may also inhibit the STAT3 signaling pathway. Treatment with aflatoxin B1 (AFB1) affects the AHR/TLR/STAT signaling axis, leading to increased AHR and TLR4 expression while downregulating STAT3 and p-STAT3 Ser727, illustrating the pathway’s complexity [106].

### 6.2. The Pro-Fibrotic Effects of AHR Ligands

AHR can be activated by structurally diverse ligands from various sources, leading to recruitment of different co-regulators and divergent biological outcomes. Environmental AHR ligands often promote fibrosis through cytotoxicity, elevated oxidative stress, and enhanced inflammation [107]. Benzo[a]pyrene (BaP) promotes collagen deposition through the NRF2 signaling pathway and exacerbates DSS-induced colitis by enhancing the expression of the pro-fibrotic protein matrix metalloproteinase-1 (MMP-1) in intestinal epithelial cells [108,109]. Dietary Trp is metabolized by gut microbiota into indole compounds, which are further metabolized by cytochrome P450 family 2 subfamily E member 1 (CYP2E1) and sulfotransferase family 1A member 1 (SULT1A1) into indoxyl sulfate (IS). IS is an endogenous AHR ligand. While IS is normally cleared renally, its accumulation during kidney failure upregulates and activates AHR, promoting PPARG coactivator 1α (PGC1α) ubiquitination and degradation, thereby accelerating renal aging and fibrosis [110].

### 6.3. AHR-Mediated Multiorgan Toxicity

AHR activation by indole compounds may also induce liver injury and nervous system effects. Flutamide for the treatment of prostate cancer causes specific hepatotoxicity. As an AHR ligand, this drug activates AHR upon administration, thereby inducing the expression of the bile acid transporter ATP-binding cassette subfamily C member 4 (ABCC4) and suppressing farnesoid X receptor (FXR) signaling. This leads to severe liver damage, including cholestasis, jaundice, and hepatic necrosis [111]. Gut dysbiosis and excessive Trp metabolite production can disrupt cerebral vascular homeostasis, impair vasodilation, induce oxidative stress and inflammation, promote cellular senescence, and ultimately compromise blood–brain barrier integrity and cognition [112]. In mouse models of chronic kidney disease (CKD) and acute kidney injury (AKI), elevated serum IS activates AHR not only in blood vessels, but also in hepatocytes and cardiomyocytes [113]. In microglia, AHR activation during ischemic pain induces oxidative stress and inflammation, causing vasogenic edema and brain injury [114].

### 6.4. Challenges in AHR-Targeted Therapy for CF

Although indole compounds could theoretically modulate CFTR via AHR, several challenges remain for direct CF therapy (Table 2). Due to variations in AHR ligand types, bioavailability, and half-lives, their efficacy and effects also differ [115]. Currently, there is a lack of clinical data on the use of indole compounds for treating CF patients. No direct evidence exists to suggest that AHR ligands, such as indole-based drugs, can be effectively delivered to key pathological sites in CF patients, such as the airway mucosa. Furthermore, their absorption may be compromised by the pathological conditions of the lungs in CF patients. Theoretical therapeutic benefits may be difficult to achieve due to low drug targeting efficiency. AHR is widely expressed throughout the body. After indole compounds activate the AHR signaling pathway, they may simultaneously affect multiple organs. While potentially improving lung function in CF patients, this could also cause adverse reactions in other tissues, such as liver toxicity. Moreover, the metabolism and clearance of AHR ligands may be altered due to changes in organ function among CF patients, leading to a mismatch between blood drug concentrations and tissue effects. The potential value of indole compounds activating the AHR signaling pathway to improve CF remains based on preclinical studies, with no clinical data available on the long-term effects of AHR activation. Particularly when administered long-term to CF patients, this may pose unknown long-term health risks, such as immunosuppression or even tumorigenesis. Rigorous and sustained clinical monitoring is required to assess its safety.

## 7. Summary and Prospect

When AHR was first discovered, it served as a receptor for environmental pollutants such as dioxins and polycyclic aromatic hydrocarbons. Researchers often avoid AHR during the drug development process. However, as research deepened, researchers gradually discovered that AHR plays an indispensable role in human physiological activities. It not only participates in maintaining the balance of the intestinal microecology but also plays a key regulatory role in the occurrence and development of inflammatory diseases. This cognitive transformation provides a new target for the treatment of complex diseases such as CF.

This review systematically sorts out the molecular pathological mechanism and clinical treatment status of CF. Focusing on the mechanism of action of the indole compounds-AHR signaling pathway, and deeply demonstrating its multiple effects in regulating pulmonary infection and inflammation, repairing intestinal barrier function, and maintaining immune homeostasis in patients with CF. For researchers developing therapeutic drugs, at the basic research level, this review has identified the core intervention nodes of the indole compounds-AHR pathway, such as antibacterial colonization, tight junction protein expression, and gut microbiota metabolic regulation, providing a direction for the design of mechanism validation experiments. At the drug development level, this study summarizes the structural characteristics, targeted delivery techniques, and preclinical data of existing indole compounds, providing references for the design of new compounds and the development of drug dosage forms. At the clinical translational level, the limitations of current research, such as tissue specificity and long-term safety, were pointed out to help researchers avoid development risks and advance novel CF therapeutics from the laboratory to the clinic.

Future research should focus on the structural screening of indole compounds, the development of nanodelivery systems, and clinical translational research. The indole compounds currently used in research are mostly broad-spectrum AHR agonists. Although they exhibit certain activities in anti-inflammation and antibacterial aspects, they may also cause liver and kidney toxicity or other side effects due to non-specific activation of AHR in multiple tissues. Therefore, researchers should deepen the study of the mechanism and clarify the specific action mechanism of AHR in different organ systems, such as the lungs and intestines of CF patients. They should also analyze the structure-activity relationship of indole compounds with different structures, and design and screen their molecular structures. A research team from Shenyang Pharmaceutical University identified indole compounds through high-throughput screening and subsequent structural optimization. These compounds demonstrated tissue-protective effects in a bleomycin-induced mouse model of pulmonary fibrosis, providing experimental evidence for the development of highly selective indole compounds [118]. Secondly, researchers should optimize the route of administration. Nanodelivery systems have become an important approach to enhancing drug targeting and therapeutic safety [119]. As mentioned above, formulating ICA into an inhalable dry powder or encapsulating it in enteric-coated microparticles can achieve targeted delivery to the lungs and intestines, significantly reducing systemic exposure and related toxicity risks. Similarly, after being nanoencapsulated, indomethacin effectively reduced its irritation to the gastric mucosa while maintaining its efficacy [120]. These cases demonstrate that nanodelivery systems are a key strategy to overcome issues such as poor targeting and low chemical stability in indole compounds. In the future, it is necessary to further develop vectors targeting different pathological features of CF to achieve more precise drug delivery. On this basis, researchers also need to strengthen clinical translational research, conduct long-term clinical trials, systematically evaluate the safety and efficacy of indole compounds as monotherapy or in combination, optimize the administration regimen, determine the optimal clinical dose range in combination with pharmacokinetic data, and explore intermittent administration patterns to reduce the risk of long-term activation of aromatic hydrocarbon receptors.

From preclinical research to safe and effective clinical drugs, the application of indole compounds targeting the AHR pathway in the treatment of CF still requires long-term exploration. With the deepening of mechanism research, the advancement of drug development technology, and the accumulation of clinical data, it may bring more precise and safer treatment options for CF patients.

## 8. Materials and Methods

A preliminary literature search on CF, indole compounds and AHR was completed through the PubMed database. The search terms include “cystic fibrosis” or “indole compounds” and “arly hydrocarbon receptor” or “indole compounds” and “drug development” or “cystic fibrosis” and “small molecule targeted drugs”. The search results were screened according to the inclusion criteria, which were English articles describing cystic fibrosis, indole compounds or aryl hydrocarbon receptors. Studies that met the above criteria were included after screening the titles, abstracts and full texts. After screening, 120 articles were finally included to further support our findings.

## Figures and Tables

**Table 2:** Potential risks and gaps. CF: cystic fibrosis. CFTR: cystic fibrosis transmembrane conductance regulator. FXR: farnesoid X receptor. CYP450: cytochrome P450. NF-κB: nuclear factor kappa-B.

Risk Category	Potential Risks	Research Gaps
Tissue specific risk	Pulmonary effects: Induce oxidative stress and exacerbate airway inflammation [116]; Liver damage: Inhibition of the FXR signaling pathway induces liver injury [111]	Lack of targeted toxicity assessment for specific organs such as the lungs, liver, and kidneys in CF patients
Exposure duration	Long-term risk: Animal studies indicate that prolonged use may induce CYP450, potentially affecting the metabolism of other drugs [117]	Lack of long-term pharmacokinetic studies in CF patients
Immunosuppression	Inhibition of the NF-κB signaling pathway in the tumor microenvironment weakens the tumor immune response and promotes tumor immune escape [104,105]	The equilibrium point between immunosuppressive effects and anti-inflammatory actions remains unclear
Drug–drug interactions	Metabolic interference: Indole compounds may affect the concentrations of other drugs by influencing the metabolic enzyme CYP450 [117]	Lack of interaction studies between indole compounds and CFTR modulators
	Drug interactions: The risk of interactions with other CF treatments such as antibiotics, bronchodilators, and CFTR modulators is unknown	Lack of research on the metabolic patterns of compounds under the unique metabolic state of CF patients
Clinical status	Existing research directions predominantly focus on cancer or inflammatory bowel disease, with insufficient preclinical data for CF patients	Lack of preclinical safety data for long-term administration

## Data Availability

No new data were created or analyzed in this study. Data sharing is not applicable to this article.

## Abbreviations

The following abbreviations are used in this manuscript:

ABC	ATP-binding cassette
ABCC4	ATP-binding cassette subfamily C member 4
AECs	Alveolar epithelial cells
AFB1	Aflatoxin B1
AHR	Aryl hydrocarbon receptor
AHRR	Aryl hydrocarbon receptor repressor
AKI	Acute kidney injury
AKT	Protein Kinase B
ARNT	Aryl hydrocarbon receptor nuclear translocator
ASL	Airway surface liquid
BAL	Bronchoalveolar lavage
BaP	Benzo[a]pyrene
bHLH	Basic helix-loop-helix
CAFs	Cancer-associated fibroblasts
CBAVD	Congenital bilateral absence of the vas deferens
CF	Cystic fibrosis
CFTR	Cystic fibrosis transmembrane conductance regulator
CKD	Chronic kidney disease
CLL	Chronic lymphocytic leukemia
CUAVD	Congenital unilateral absence of the vas deferens
CYP1A1	Cytochrome P450 family 1 subfamily A member 1
CYP1B1	Cytochrome P450 family 1 subfamily B member 1
CYP2E1	Cytochrome P450 family 2 subfamily E member 1
DCs	Dendritic cells
DIM	3,3′-diindolylmethane
DSS	Dextran sulfate sodium salt
ENaC	Epithelial sodium channel
ER	Endoplasmic reticulum
ERK	Extracellular regulated protein kinase
ERQC	Endoplasmic reticulum quality control
FICZ	6-formylindole [3,2b]carbazole
FXR	Farnesoid X receptor
HSP	Heat shock protein
I3C	Indole-3-carbinol
IA	Indole-3-acrylic acid
IAA	Indole-3-acetic acid
IBD	Inflammatory bowel disease
ICA	Indole-3-carbaldehyde
IFN-α	Interferon-α
IL	Interleukin
ILA	Indole-3-lactic acid
ILC3s	Type 3 innate lymphoid cells
IPA	Indole-3-propionic acid
IS	Indoxyl sulfate
ITE	2-(1′-H-indole-3′-carbonyl) -thiazole-4-carboxylic acid methyl ester
JAK2	Janus kinase 2
Kyn	Kynurenine
LPS	Lipopolysaccharide
MMP-1	Matrix metalloproteinase-1
MSD	Membrane-spanning domain
MUC	Mucin
NBD	Nucleotide-binding domain
NF-κB	Nuclear factor kappa-B
NRF2	Nuclear factor erythroid 2-related factor 2
PAS	Per-ARNT-Sim
PGC1α	PPARG coactivator 1 α
R domain	Regulatory domain
RELMβ	Resistin-like molecule β
ROS	Reactive oxygen species
RSV	Respiratory syncytial virus
SOCS	Suppressor of cytokine signaling
SR1	Stemregenin 1
STAT	Signal transducer and activator of transcription
SULT1A1	Sulfotransferase family 1A member 1
TCDD	2,3,7,8-tetrachlorodibenzo-p-dioxin
TEER	Transepithelial electrical resistance
TFF3	Trefoil factor 3
TGF-β	Transforming growth factor-β
Th	T helper cell
TJs	Tight junctions
TLR	Toll-like receptor
TNF	Tumor necrosis factor
Tregs	Regulatory cells
Trp	Tryptophan
XAP2	X-associated protein 2

## References

[1] Elias A.E., McBain A.J., Aldehalan F.A., Taylor G., O’Neill C.A. (2024). Activation of the aryl hydrocarbon receptor via indole derivatives is a common feature in skin bacterial isolates. J. Appl. Microbiol..

[2] Abu-Hashem A.A., Al-Hussain S.A. (2022). Design, Synthesis of New 1,2,4-Triazole/1,3,4-Thiadiazole with Spiroindoline, Imidazo[4,5-b]quinoxaline and Thieno[2,3-d]pyrimidine from Isatin Derivatives as Anticancer Agents. Molecules.

[3] Levring J., Terry D.S., Kilic Z., Fitzgerald G., Blanchard S.C., Chen J. (2023). CFTR function, pathology and pharmacology at single-molecule resolution. Nature.

[4] Kleinfelder K., Somenza E., Farinazzo A., Conti J., Lotti V., Latorre R.V., Rodella L., Massella A., Tomba F., Bertini M. (2023). CFTR Modulators Rescue the Activity of CFTR in Colonoids Expressing the Complex Allele p.[R74W;V201M;D1270N]/dele22_24. Int. J. Mol. Sci..

[5] Angyal D., Kleinfelder K., Ciciriello F., Groeneweg T.A., De Marchi G., de Pretis N., Bernardoni L., Rodella L., Tomba F., De Angelis P. (2024). CFTR function is impaired in a subset of patients with pancreatitis carrying rare CFTR variants. Pancreatology.

[6] Ébert A., Gál E., Tóth E., Szögi T., Hegyi P., Venglovecz V. (2024). Role of CFTR in diabetes-induced pancreatic ductal fluid and HCO_3_^−^ secretion. J. Physiol..

[7] Luan X., Henao Romero N., Campanucci V.A., Le Y., Mustofa J., Tam J.S., Ianowski J.P. (2024). Pulmonary Ionocytes Regulate Airway Surface Liquid pH in Primary Human Bronchial Epithelial Cells. Am. J. Respir. Crit. Care Med..

[8] Xie Y., Lu L., Tang X.X., Moninger T.O., Huang T.J., Stoltz D.A., Welsh M.J. (2020). Acidic Submucosal Gland pH and Elevated Protein Concentration Produce Abnormal Cystic Fibrosis Mucus. Dev. Cell..

[9] Hanssens L.S., Duchateau J., Casimir G.J. (2021). CFTR Protein: Not Just a Chloride Channel?. Cells.

[10] Anderson K.J., Cormier R.T., Scott P.M. (2019). Role of ion channels in gastrointestinal cancer. World J. Gastroenterol..

[11] Myerburg M.M., Butterworth M.B., McKenna E.E., Peters K.W., Frizzell R.A., Kleyman T.R., Pilewski J.M. (2006). Airway surface liquid volume regulates ENaC by altering the serine protease-protease inhibitor balance: A mechanism for sodium hyperabsorption in cystic fibrosis. J. Biol. Chem..

[12] Montoro D.T., Haber A.L., Biton M., Vinarsky V., Lin B., Birket S.E., Yuan F., Chen S., Leung H.M., Villoria J. (2018). A revised airway epithelial hierarchy includes CFTR-expressing ionocytes. Nature.

[13] Zhou W., Wang Y., Yang Y., Sun Y., Cheng C., Dai J., Meng S., Chen K., Zhao Y., Liu X. (2025). Progression and mortality of patients with cystic fibrosis in China. Orphanet J. Rare Dis..

[14] Grünewaldt A., Rohde G. (2025). Retrospective cohort study of adult patients with cystic fibrosis supported with venovenous extracorporeal membrane oxygenation (VV ECMO) at a large German cystic fibrosis center. BMC Pulm. Med..

[15] de Macedo J.R.F.F., Aubriot A.S., Reychler G., Penelle M., Gohy S., Poncin W. (2024). The intermittent intrapulmonary deflation technique for airway clearance in patients with cystic fibrosis: A randomized trial. Respir. Med. Res..

[16] Long A.M., Jones I.H., Knight M., McNally J. (2021). BAPS-CASS Early management of meconium ileus in infants with cystic fibrosis: A prospective population cohort study. J. Pediatr. Surg..

[17] Gao X., Yeh H.I., Yang Z., Fan C., Jiang F., Howard R.J., Lindahl E., Kappes J.C., Hwang T.C. (2024). Allosteric inhibition of CFTR gating by CFTRinh-172 binding in the pore. Nat. Commun..

[18] Infield D.T., Strickland K.M., Gaggar A., McCarty N.A. (2021). The molecular evolution of function in the CFTR chloride channel. J. Gen. Physiol..

[19] Cannarella R., Leitner D.V., Weiss M., Vij S.C. (2025). Testicular function and fertility outcomes in males with CF: A multi center retrospective study of men with congenital bilateral absence of the vas deferens based on CFTR mutation status. J. Clin. Transl. Endocrinol..

[20] Jathal I., Stransky O.M., Wright C.E., Prangley A., Tangpricha V., Jain R., Taylor-Cousar J.L., Hughan K.S., Ladores-Barrett S., West N.E. (2025). Fertility and family-building experiences and perspectives of males with cystic fibrosis. Reprod. Biol. Endocrinol..

[21] Borgo C., D’Amore C., Capurro V., Tomati V., Pedemonte N., Bosello Travain V., Salvi M. (2024). SUMOylation Inhibition Enhances Protein Transcription under CMV Promoter: A Lesson from a Study with the F508del-CFTR Mutant. Int. J. Mol. Sci..

[22] Guhr Lee T.N., Cholon D.M., Quinney N.L., Gentzsch M., Esther C.R. (2020). Accumulation and persistence of ivacaftor in airway epithelia with prolonged treatment. J. Cyst. Fibros..

[23] Amaral M.D., Farinha C.M., Matos P., Botelho H.M. (2016). Investigating Alternative Transport of Integral Plasma Membrane Proteins from the ER to the Golgi: Lessons from the Cystic Fibrosis Transmembrane Conductance Regulator (CFTR). Methods Mol. Biol..

[24] Elborn J.S. (2016). Cystic fibrosis. Lancet.

[25] McDonald E.F., Woods H., Smith S.T., Kim M., Schoeder C.T., Plate L., Meiler J. (2022). Structural Comparative Modeling of Multi-Domain F508del CFTR. Biomolecules.

[26] Thavamani A., Salem I., Sferra T.J., Sankararaman S. (2021). Impact of Altered Gut Microbiota and Its Metabolites in Cystic Fibrosis. Metabolites.

[27] He R., Lin F., Deng Z., Yu B. (2024). Elexacaftor-tezacaftor-ivacaftor for cystic fibrosis with Phe508del mutation: Evidence from randomized controlled trials. SAGE Open Med..

[28] Meng X., Ford R.C. (2024). Investigation of F508del CFTR unfolding and a search for stabilizing small molecules. Arch. Biochem. Biophys..

[29] Merlo C., Thorat T., McGarry L.J., Scirica C.V., DerSarkissian M., Nguyen C., Gu Y.M., Muthukumar A., Healy J., Rubin J.L. (2024). A Retrospective, Longitudinal Registry Study on the Long-Term Durability of Ivacaftor Treatment in People with Cystic Fibrosis. Pulm. Ther..

[30] Drummond D., Dana J., Berteloot L., Schneider-Futschik E.K., Chedevergne F., Bailly-Botuha C., Nguyen-Khoa T., Cornet M., Le Bourgeois M., Debray D. (2022). Lumacaftor-ivacaftor effects on cystic fibrosis-related liver involvement in adolescents with homozygous F508 del-CFTR. J. Cyst. Fibros..

[31] Bacalhau M., Camargo M., Magalhães-Ghiotto G.A.V., Drumond S., Castelletti C.H.M., Lopes-Pacheco M. (2023). Elexacaftor-Tezacaftor-Ivacaftor: A Life-Changing Triple Combination of CFTR Modulator Drugs for Cystic Fibrosis. Pharmaceuticals.

[32] Dai S., Qu L., Li J., Zhang Y., Jiang L., Wei H., Guo M., Chen X., Chen Y. (2022). Structural insight into the ligand binding mechanism of aryl hydrocarbon receptor. Nat. Commun..

[33] Wen Z., Zhang Y., Zhang B., Hang Y., Xu L., Chen Y., Xie Q., Zhao Q., Zhang L., Li G. (2023). Cryo-EM structure of the cytosolic AhR complex. Structure.

[34] Polonio C.M., McHale K.A., Sherr D.H., Rubenstein D., Quintana F.J. (2025). The aryl hydrocarbon receptor: A rehabilitated target for therapeutic immune modulation. Nat. Rev. Drug Discov..

[35] Sondermann N.C., Faßbender S., Hartung F., Hätälä A.M., Rolfes K.M., Vogel C.F.A., Haarmann-Stemmann T. (2023). Functions of the aryl hydrocarbon receptor (AHR) beyond the canonical AHR/ARNT signaling pathway. Biochem. Pharmacol..

[36] Kobayashi S., Okamoto H., Iwamoto T., Toyama Y., Tomatsu T., Yamanaka H., Momohara S. (2008). A role for the aryl hydrocarbon receptor and the dioxin TCDD in rheumatoid arthritis. Rheumatology.

[37] You Y., Cai B., Zhu C., Zhou Z., Xu J., Huang L., Jie L., Du H. (2025). 3,3′-diindolylmethane, from cruciferous vegetables, ameliorates cigarette smoke-induced inflammatory amplification in CIA model mice by targeting the AhR/NF-κB crosstalk. J. Nutr. Biochem..

[38] Sawa-Wejksza K., Parada-Turska J., Turski W. (2025). The Pharmacological Evidences for the Involvement of AhR and GPR35 Receptors in Kynurenic Acid-Mediated Cytokine and Chemokine Secretion by THP-1-Derived Macrophages. Molecules.

[39] Ishihara Y., Kado S.Y., Bein K.J., He Y., Pouraryan A.A., Urban A., Haarmann-Stemmann T., Sweeney C., Vogel C.F.A. (2022). Aryl Hydrocarbon Receptor Signaling Synergizes with TLR/NF-κB-Signaling for Induction of IL-22 Through Canonical and Non-Canonical AhR Pathways. Front. Toxicol..

[40] Wang Y.N., Wu X., Shan Q.Y., Yang Q., Yu X.Y., Yang J.H., Miao H., Cao G., Zhao Y.Y. (2025). Acteoside-containing caffeic acid is bioactive functional group of antifibrotic effect by suppressing inflammation via inhibiting AHR nuclear translocation in chronic kidney disease. Acta Pharmacol. Sin..

[41] Xiong J., Zhang X., Zhang Y., Wu B., Fang L., Wang N., Yi H., Chang N., Chen L., Zhang J. (2020). Aryl hydrocarbon receptor mediates Jak2/STAT3 signaling for non-small cell lung cancer stem cell maintenance. Exp. Cell Res..

[42] Zhang Y., Han L., Dong J., Yuan Z., Yao W., Ji P., Hua Y., Wei Y. (2024). Shaoyao decoction improves damp-heat colitis by activating the AHR/IL-22/STAT3 pathway through tryptophan metabolism driven by gut microbiota. J. Ethnopharmacol..

[43] Tsai C.H., Lee Y., Li C.H., Cheng Y.W., Kang J.J. (2020). Down-regulation of aryl hydrocarbon receptor intensifies carcinogen-induced retinal lesion via SOCS3-STAT3 signaling. Cell Biol. Toxicol..

[44] Wang L., Cheng H., Wang X., Zhu F., Tian N., Xu Z., Yin H., Liang M., Yang X., Liu X. (2024). Deubiquitination of aryl hydrocarbon receptor by USP21 negatively regulates T helper 17 cell differentiation. J. Leukoc. Biol..

[45] Dvořák Z., Mani S., Vondráček J. (2025). Emerging approaches for antagonizing the aryl hydrocarbon receptor. Trends Pharmacol. Sci..

[46] Veland N., Gleneadie H.J., Brown K.E., Sardini A., Pombo J., Dimond A., Burns V., Sarkisyan K., Schiering C., Webster Z. (2024). Bioluminescence imaging of Cyp1a1-luciferase reporter mice demonstrates prolonged activation of the aryl hydrocarbon receptor in the lung. Commun. Biol..

[47] Zhou L., Song W., Liu T., Yan T., He Z., He W., Lv J., Zhang S., Dai X., Yuan L. (2024). Multi-omics insights into anti-colitis benefits of the synbiotic and postbiotic derived from wheat bran arabinoxylan and *Limosilactobacillus reuteri*. Int. J. Biol. Macromol..

[48] Dutta B., Tripathy A., Archana P.R., Kamath S.U. (2025). Unraveling the complexities of diet induced obesity and glucolipid dysfunction in metabolic syndrome. Diabetol. Metab. Syndr..

[49] Dvořák Z., Poulíková K., Mani S. (2021). Indole scaffolds as a promising class of the aryl hydrocarbon receptor ligands. Eur. J. Med. Chem..

[50] Wang B., Sun S., Liu M., Chen H., Liu N., Wu Z., Wu G., Dai Z. (2020). Dietary L-Tryptophan Regulates Colonic Serotonin Homeostasis in Mice with Dextran Sodium Sulfate-Induced Colitis. J. Nutr..

[51] Roager H.M., Licht T.R. (2018). Microbial tryptophan catabolites in health and disease. Nat. Commun..

[52] Debnath B., Nandi B., Paul S., Manna S., Maity A., Bandyopadhyay K., Panda S., Khan S., Nath R., Akhtar M. (2025). Novel indole-based synthetic molecules in cancer treatment: Synthetic strategies and structure-activity relationship. Med. Drug Discov..

[53] Xue C., Li G., Zheng Q., Gu X., Shi Q., Su Y., Chu Q., Yuan X., Bao Z., Lu J. (2023). Tryptophan metabolism in health and disease. Cell Metab..

[54] Yang J., Wang H., Yan J., Sun J., Wang Y., Huang G., Zhang F., Cao H., Li D. (2025). Biotherapeutic potential of gut microbiota-derived indole-3-acetic acid. Crit. Rev. Microbiol..

[55] Zelante T., Iannitti R.G., Cunha C., De Luca A., Giovannini G., Pieraccini G., Zecchi R., D’Angelo C., Massi-Benedetti C., Fallarino F. (2013). Tryptophan catabolites from microbiota engage aryl hydrocarbon receptor and balance mucosal reactivity via interleukin-22. Immunity.

[56] Hou Q., Ye L., Liu H., Huang L., Yang Q., Turner J.R., Yu Q. (2018). Lactobacillus accelerates ISCs regeneration to protect the integrity of intestinal mucosa through activation of STAT3 signaling pathway induced by LPLs secretion of IL-22. Cell Death Differ..

[57] Ma Y., Wang Q., Yu K., Fan X., Xiao W., Cai Y., Xu P., Yu M., Yang H. (2018). 6-Formylindolo(3,2-b)carbazole induced aryl hydrocarbon receptor activation prevents intestinal barrier dysfunction through regulation of claudin-2 expression. Chem. Biol. Interact..

[58] Agus A., Planchais J., Sokol H. (2018). Gut Microbiota Regulation of Tryptophan Metabolism in Health and Disease. Cell Host Microbe.

[59] Jonić N., Koprivica I., Chatzigiannis C.M., Tsiailanis A.D., Kyrkou S.G., Tzakos E.P., Pavić A., Dimitrijević M., Jovanović A., Jovanović M.B. (2024). Development of FluoAHRL: A Novel Synthetic Fluorescent Compound That Activates AHR and Potentiates Anti-Inflammatory T Regulatory Cells. Molecules.

[60] Wellems D., Hu Y., Jennings S., Wang G. (2023). Loss of CFTR function in macrophages alters the cell transcriptional program and delays lung resolution of inflammation. Front. Immunol..

[61] Fan Y., Pedersen O. (2021). Gut microbiota in human metabolic health and disease. Nat. Rev. Microbiol..

[62] Greenwald M.A., Wolfgang M.C. (2022). The changing landscape of the cystic fibrosis lung environment: From the perspective of *Pseudomonas aeruginosa*. Curr. Opin. Pharmacol..

[63] Vohra M., Kamath N., Dubey R., Sharma S., Sharma S. (2025). Complex Interplay between Biofilm Formation, Antibiotic Resistance, and Virulence in *Pseudomonas aeruginosa*: A Phenotypic and Genotypic Study. Mol. Genet. Microbiol. Virol..

[64] Moura-Alves P., Puyskens A., Stinn A., Klemm M., Guhlich-Bornhof U., Dorhoi A., Furkert J., Kreuchwig A., Protze J., Lozza L. (2019). Host monitoring of quorum sensing during *Pseudomonas aeruginosa* infection. Science.

[65] Puccetti M., Gomes Dos Reis L., Pariano M., Costantini C., Renga G., Ricci M., Traini D., Giovagnoli S. (2021). Development and in vitro-in vivo performances of an inhalable indole-3-carboxaldehyde dry powder to target pulmonary inflammation and infection. Int. J. Pharm..

[66] Britto C.J., Brady V., Lee S., Dela Cruz C.S. (2017). Respiratory Viral Infections in Chronic Lung Diseases. Clin. Chest Med..

[67] Major J., Crotta S., Finsterbusch K., Chakravarty P., Shah K., Frederico B., D’Antuono R., Green M., Meader L., Suarez-Bonnet A. (2023). Endothelial AHR activity prevents lung barrier disruption in viral infection. Nature.

[68] Zimmerman E., Sturrock A., Reilly C.A., Burrell-Gerbers K.L., Warren K., Mir-Kasimov M., Zhang M.A., Pierce M.S., Helms M.N., Paine R. (2024). Aryl Hydrocarbon Receptor Activation in Pulmonary Alveolar Epithelial Cells Limits Inflammation and Preserves Lung Epithelial Cell Integrity. J. Immunol..

[69] Aljameeli A.M., Alsuwayt B., Bharati D., Gohri V., Mohite P., Singh S., Chidrawar V. (2025). Chloride channels and mast cell function: Pioneering new frontiers in IBD therapy. Mol. Cell Biochem..

[70] Duong J.T., Hayden H.S., Verster A.J., Pope C.E., Miller C., Penewit K., Salipante S.J., Rowe S.M., Solomon G.M., Nichols D. (2025). Fecal microbiota changes in people with cystic fibrosis after 6 months of elexacaftor/tezacaftor/ivacaftor: Findings from the promise study. J. Cyst. Fibros..

[71] Wang G., Fan Y., Zhang G., Cai S., Ma Y., Yang L., Wang Y., Yu H., Qiao S., Zeng X. (2024). Microbiota-derived indoles alleviate intestinal inflammation and modulate microbiome by microbial cross-feeding. Microbiome.

[72] Park S.L., Justiniano R., Williams J.D., Cabello C.M., Qiao S., Wondrak G.T. (2015). The Tryptophan-Derived Endogenous Aryl Hydrocarbon Receptor Ligand 6-Formylindolo[3,2-b]Carbazole Is a Nanomolar UVA Photosensitizer in Epidermal Keratinocytes. J. Investig. Dermatol..

[73] Liu D., Zhang S., Li S., Zhang Q., Cai Y., Li P., Li H., Shen B., Liao Q., Hong Y. (2023). Indoleacrylic acid produced by Parabacteroides distasonis alleviates type 2 diabetes via activation of AhR to repair intestinal barrier. BMC Biol..

[74] de Vrankrijker A.M., Wolfs T.F., van der Ent C.K. (2010). Challenging and emerging pathogens in cystic fibrosis. Paediatr. Respir. Rev..

[75] Huang C., Song P., Fan P., Hou C., Thacker P., Ma X. (2015). Dietary Sodium Butyrate Decreases Postweaning Diarrhea by Modulating Intestinal Permeability and Changing the Bacterial Communities in Weaned Piglets. J. Nutr..

[76] Durda-Masny M., Goździk-Spychalska J., Morańska K., Pawłowska N., Mazurkiewicz M., Skrzypczak I., Cofta S., Szwed A. (2024). Gut microbiota in adults with cystic fibrosis: Implications for the severity of the CFTR gene mutation and nutritional status. J. Cyst. Fibros..

[77] Hayden H.S., Eng A., Pope C.E., Brittnacher M.J., Vo A.T., Weiss E.J., Hager K.R., Martin B.D., Leung D.H., Heltshe S.L. (2020). Fecal dysbiosis in infants with cystic fibrosis is associated with early linear growth failure. Nat. Med..

[78] Sun M., Ma N., He T., Johnston L.J., Ma X. (2020). Tryptophan (Trp) modulates gut homeostasis via aryl hydrocarbon receptor (AhR). Crit. Rev. Food Sci. Nutr..

[79] Lu H., Zhang G., Wang K., Liu K., Gao Y., Chen J., Li Y., Yan J. (2025). The Role of Lactiplantibacillus plantarum CGMCC9513 in Alleviating Colitis by Synergistic Enhancement of the Intestinal Barrier Through Modulating Gut Microbiota and Activating the Aryl Hydrocarbon Receptor. Probiotics Antimicrob Proteins.

[80] Jaudas F., Bartenschlager F., Shashikadze B., Santamaria G., Reichart D., Schnell A., Stöckl J.B., Degroote R.L., Cambra J.M., Graeber S.Y. (2025). Perinatal dysfunction of innate immunity in cystic fibrosis. Sci. Transl. Med..

[81] Koeppen K., Nymon A., Barnaby R., Li Z., Hampton T.H., Ashare A., Stanton B.A. (2021). CF monocyte-derived macrophages have an attenuated response to extracellular vesicles secreted by airway epithelial cells. Am. J. Physiol. Lung Cell Mol. Physiol..

[82] Goudot C., Coillard A., Villani A.C., Gueguen P., Cros A., Sarkizova S., Tang-Huau T.L., Bohec M., Baulande S., Hacohen N. (2017). Aryl Hydrocarbon Receptor Controls Monocyte Differentiation into Dendritic Cells versus Macrophages. Immunity.

[83] Hou X., Zhang X., Bi J., Zhu A., He L. (2021). Indole-3-carboxaldehyde regulates RSV-induced inflammatory response in RAW264.7 cells by moderate inhibition of the TLR7 signaling pathway. J. Nat. Med..

[84] Piñero-Fernandez S., Chimerel C., Keyser U.F., Summers D.K. (2011). Indole transport across Escherichia coli membranes. J. Bacteriol..

[85] Jing W., Dong S., Xu Y., Liu J., Ren J., Liu X., Zhu M., Zhang M., Shi H., Li N. (2025). Gut microbiota-derived tryptophan metabolites regulated by Wuji Wan to attenuate colitis through AhR signaling activation. Acta Pharm. Sin. B.

[86] Li J., Zhang L., Wu T., Li Y., Zhou X., Ruan Z. (2021). Indole-3-propionic Acid Improved the Intestinal Barrier by Enhancing Epithelial Barrier and Mucus Barrier. J. Agric. Food Chem..

[87] Gao H., Sun M., Li A., Gu Q., Kang D., Feng Z., Li X., Wang X., Chen L., Yang H. (2025). Microbiota-derived IPA alleviates intestinal mucosal inflammation through upregulating Th1/Th17 cell apoptosis in inflammatory bowel disease. Gut Microbes.

[88] Munakarmi S., Chand L., Shin H.B., Jang K.Y., Jeong Y.J. (2020). Indole-3-Carbinol Derivative DIM Mitigates Carbon Tetrachloride-Induced Acute Liver Injury in Mice by Inhibiting Inflammatory Response, Apoptosis and Regulating Oxidative Stress. Int. J. Mol. Sci..

[89] Fu Y., Gao H., Hou X., Chen Y., Xu K. (2022). Pretreatment with IPA ameliorates colitis in mice: Colon transcriptome and fecal 16S amplicon profiling. Front. Immunol..

[90] Choi Y., Abdelmegeed M.A., Song B.J. (2018). Preventive effects of indole-3-carbinol against alcohol-induced liver injury in mice via antioxidant, anti-inflammatory, and anti-apoptotic mechanisms: Role of gut-liver-adipose tissue axis. J. Nutr. Biochem..

[91] Puccetti M., Pariano M., Renga G., Santarelli I., D’Onofrio F., Bellet M.M., Stincardini C., Bartoli A., Costantini C., Romani L. (2021). Targeted Drug Delivery Technologies Potentiate the Overall Therapeutic Efficacy of an Indole Derivative in a Mouse Cystic Fibrosis Setting. Cells.

[92] Ding Y., Yanagi K., Yang F., Callaway E., Cheng C., Hensel M.E., Menon R., Alaniz R.C., Lee K., Jayaraman A. (2024). Oral supplementation of gut microbial metabolite indole-3-acetate alleviates diet-induced steatosis and inflammation in mice. eLife.

[93] Wlodarska M., Luo C., Kolde R., d’Hennezel E., Annand J.W., Heim C.E., Krastel P., Schmitt E.K., Omar A.S., Creasey E.A. (2017). Indoleacrylic Acid Produced by Commensal Peptostreptococcus Species Suppresses Inflammation. Cell Host Microbe.

[94] Genestet C., Le Gouellec A., Chaker H., Polack B., Guery B., Toussaint B., Stasia M.J. (2014). Scavenging of reactive oxygen species by tryptophan metabolites helps *Pseudomonas aeruginosa* escape neutrophil killing. Free Radic. Biol. Med..

[95] Mustafina M., Silantyev A., Krasovskiy S., Chernyak A., Naumenko Z., Suvorov A., Gognieva D., Abdullaev M., Bektimirova A., Bykova A. (2024). Exhaled breath analysis in adult patients with cystic fibrosis by real-time proton mass spectrometry. Clin. Chim. Acta.

[96] Mustafina M., Silantyev A., Krasovskiy S., Chernyak A., Naumenko Z., Suvorov A., Gognieva D., Abdullaev M., Suvorova O., Schmidt A. (2024). Identification of Exhaled Metabolites Correlated with Respiratory Function and Clinical Features in Adult Patients with Cystic Fibrosis by Real-Time Proton Mass Spectrometry. Biomolecules.

[97] Puccetti M., Pariano M., Wojtylo P., Schoubben A., Giovagnoli S., Ricci M. (2023). Turning Microbial AhR Agonists into Therapeutic Agents via Drug Delivery Systems. Pharmaceutics.

[98] Rueda G.H., Causada-Calo N., Borojevic R., Nardelli A., Pinto-Sanchez M.I., Constante M., Libertucci J., Mohan V., Langella P., Loonen L.M.P. (2024). Oral tryptophan activates duodenal aryl hydrocarbon receptor in healthy subjects: A crossover randomized controlled trial. Am. J. Physiol. Gastrointest. Liver Physiol..

[99] Zeng W., Han C., Mohammed S., Li S., Song Y., Sun F., Du Y. (2024). Indole-containing pharmaceuticals: Targets, pharmacological activities, and SAR studies. RSC Med. Chem..

[100] Fiedorczuk K., Chen J. (2022). Mechanism of CFTR correction by type I folding correctors. Cell.

[101] Ruah S.S.H., Grootenhuis P.D., Van Goor F., Zhou J., Bear B., Miller M.T., Numa M.M.D., Yang X. (2025). Indole Derivatives as CFTR Modulators. U.S. Patent.

[102] Son J.H., Phuan P.W., Zhu J.S., Lipman E., Cheung A., Tsui K.Y., Tantillo D.J., Verkman A.S., Haggie P.M., Kurth M.J. (2021). 1-BENZYLSPIRO[PIPERIDINE-4,1′-PYRIDO[3,4-*b]indole*] ‘co-potentiators’ for minimal function CFTR mutants. Eur. J. Med. Chem..

[103] Brindani N., Gianotti A., Giovani S., Giacomina F., Di Fruscia P., Sorana F., Bertozzi S.M., Ottonello G., Goldoni L., Penna I. (2020). Identification, Structure-Activity Relationship, and Biological Characterization of 2,3,4,5-Tetrahydro-1*H*-pyrido[4,3-*b*]indoles as a Novel Class of CFTR Potentiators. J. Med. Chem..

[104] Sadik A., Somarribas Patterson L.F., Öztürk S., Mohapatra S.R., Panitz V., Secker P.F., Pfänder P., Loth S., Salem H., Prentzell M.T. (2020). IL4I1 Is a Metabolic Immune Checkpoint that Activates the AHR and Promotes Tumor Progression. Cell.

[105] Chen L.B., Zhu S.P., Liu T.P., Zhao H., Chen P.F., Duan Y.J., Hu R. (2021). Cancer Associated Fibroblasts Promote Renal Cancer Progression Through a TDO/Kyn/AhR Dependent Signaling Pathway. Front. Oncol..

[106] Zhang L., Cheng D., Zhang J., Tang H., Li F., Peng Y., Duan X., Meng E., Zhang C., Zeng T. (2023). Role of macrophage AHR/TLR4/STAT3 signaling axis in the colitis induced by non-canonical AHR ligand aflatoxin B1. J. Hazard. Mater..

[107] Pan Y., Deng Y., Yang H., Yu M. (2025). The aryl hydrocarbon receptor: A promising target for intestinal fibrosis therapy. Pharmacol. Res..

[108] Li M.D., Chen L.H., Xiang H.X., Jiang Y.L., Lv B.B., Xu D.X., Zhao H., Fu L. (2024). Benzo[a]pyrene evokes epithelial-mesenchymal transition and pulmonary fibrosis through AhR-mediated Nrf2-p62 signaling. J. Hazard. Mater..

[109] Luo M., Luo D., Liu J., Wang H., Liu X., Yang M., Tian F., Qin S., Li Y. (2022). Ameliorative effect of the probiotic peptide against benzo(α)pyrene-induced inflammatory damages in enterocytes. Int. Immunopharmacol..

[110] Xie H., Yang N., Lu L., Sun X., Li J., Wang X., Guo H., Zhou L., Liu J., Wu H. (2024). Uremic Toxin Receptor AhR Facilitates Renal Senescence and Fibrosis via Suppressing Mitochondrial Biogenesis. Adv. Sci..

[111] Gao X., Xie C., Wang Y., Luo Y., Yagai T., Sun D., Qin X., Krausz K.W., Gonzalez F.J. (2016). The antiandrogen flutamide is a novel aryl hydrocarbon receptor ligand that disrupts bile acid homeostasis in mice through induction of Abcc4. Biochem. Pharmacol..

[112] Salminen A. (2023). Activation of aryl hydrocarbon receptor (AhR) in Alzheimer’s disease: Role of tryptophan metabolites generated by gut host-microbiota. J. Mol. Med..

[113] Walker J.A., Richards S., Belghasem M.E., Arinze N., Yoo S.B., Tashjian J.Y., Whelan S.A., Lee N., Kolachalama V.B., Francis J. (2020). Temporal and tissue-specific activation of aryl hydrocarbon receptor in discrete mouse models of kidney disease. Kidney Int..

[114] Tanaka M., Fujikawa M., Oguro A., Itoh K., Vogel C.F.A., Ishihara Y. (2021). Involvement of the Microglial Aryl Hydrocarbon Receptor in Neuroinflammation and Vasogenic Edema after Ischemic Stroke. Cells.

[115] Moriguchi T., Motohashi H., Hosoya T., Nakajima O., Takahashi S., Ohsako S., Aoki Y., Nishimura N., Tohyama C., Fujii-Kuriyama Y. (2003). Distinct response to dioxin in an arylhydrocarbon receptor (AHR)-humanized mouse. Proc. Natl. Acad. Sci. USA.

[116] Shen Q., Yu H., Liu Y., Li G., An T. (2024). Combined exposure of MAHs and PAHs enhanced amino acid and lipid metabolism disruption in epithelium leading asthma risk. Environ. Pollut..

[117] Xu Z.Y., Li J.L. (2019). Comparative review of drug-drug interactions with epidermal growth factor receptor tyrosine kinase inhibitors for the treatment of non-small-cell lung cancer. Onco Targets Ther..

[118] Lei H., Guo M., Li X., Jia F., Li C., Yang Y., Cao M., Jiang N., Ma E., Zhai X. (2020). Discovery of Novel Indole-Based Allosteric Highly Potent ATX Inhibitors with Great In Vivo Efficacy in a Mouse Lung Fibrosis Model. J. Med. Chem..

[119] Qi Y., Yang B., Ouyang H., Wang X., Li C., Li L., Zhang J. (2025). Advanced nanotherapies for precision treatment of inflammatory lung diseases. Bioact. Mater..

[120] Wang X., Wang M., Wang Q., Yuan Y., Hao Q., Bi Y., He Y., Zhao J., Hao J. (2022). Fabrication and in vitro/in vivo characterization of Eudragit enteric nanoparticles loaded with indomethacin. Chem. Pap..

